# Physiologically based modelling of the antiplatelet effect of aspirin: A tool to characterize drug responsiveness and inform precision dosing

**DOI:** 10.1371/journal.pone.0268905

**Published:** 2022-08-17

**Authors:** Alberto Giaretta, Giovanna Petrucci, Bianca Rocca, Gianna Maria Toffolo

**Affiliations:** 1 Department of Information Engineering, University of Padova, Padova, Italy; 2 Department of Pathology, University of Cambridge, Cambridge, United Kingdom; 3 Department of Pharmacology, Catholic University School of Medicine, Rome, Italy; University of Alcalá, SPAIN

## Abstract

A computational approach involving mathematical modeling and *in silico* experiments was used to characterize the determinants of extent and duration of platelet cyclooxygenase (COX)-1 inhibition by aspirin and design precision dosing in patients with accelerated platelet turnover or reduced drug bioavailability. To this purpose, a recently developed physiologically-based pharmacokinetics (PK) and pharmacodynamics (PD) model of low-dose aspirin in regenerating platelets and megakaryocytes, was used to predict the main features and determinants of platelet COX-1 inhibition. The response to different aspirin regimens in healthy subjects and in pathological conditions associated with alterations in aspirin PK (i.e., severely obese subjects) or PD (i.e., essential thrombocytemya patients), were simulated. A model sensitivity analysis was performed to identify the main processes influencing COX-1 dynamics. *In silico* experiments and sensitivity analyses indicated a major role for megakaryocytes and platelet turnover in determining the extent and duration of COX-1 inhibition by once-daily, low-dose aspirin. They also showed the superiority of reducing the dosing interval *vs* increasing the once-daily dose in conditions of increased platelet turnover, while suggested specific dose adjustments in conditions of possible reduction in drug bioavailability. In conclusion, the consistency of our model-based findings with experimental data from studies in healthy subjects and patients with essential thrombocythemia supports the potential of our approach for describing the determinants of platelet inhibition by aspirin and informing precision dosing which may guide personalized antithrombotic therapy in different patient populations, especially in those under-represented in clinical trials or in those associated with poor feasibility.

## Introduction

Mathematical modelling and simulations are potentially useful tools in all steps of drug development and regulatory process [1]. In particular, physiologically based (PB) models, aiming at describing mechanistic processes rather than reproducing the concentration-effect relationship with empirical formulas, offer major advantages to address complex clinical and pharmacological settings [2–4]. They also provide a useful tool to predict the response to diverse therapeutic regimens across different patient populations and diseases, leading to the so called model–informed precision dosing. This approach is particularly relevant to the special populations (*i*.*e*. children, pregnancy, severely-obese subjects, elderly), to rare diseases, and complex clinical scenarios combining the above conditions, where randomized clinical trials (RCTs) are lacking, subgroup analyses are largely underpowered, or RCTs are often unfeasible [5–7].

Low-dose aspirin is the cornerstone of antiplatelet therapy for preventing and treating aterothrombotic complications in different, high-risk clinical settings. The mechanism of action of low-dose aspirin relies on permanent inactivation of the COX activity of platelet prostaglandin (PG)G/H synthase-1 (colloquially referred to as COX-1), as a consequence of the acetylation of a critical serine residue within the COX-1 channel [8–10]. In the present study, we applied a previously described PB model of the antiplatelet effect of low-dose aspirin [11] to characterize the determinants of the extent and duration of platelet cyclooxygenase (COX)-1 inhibition to help designing precision dosing regimens in patients with accelerated platelet turnover or reduced drug bioavailability. The model reproduces the long-lasting antiplatelet effect of aspirin, allowing for a once-daily dosing regimen despite its 20-min half-life, that results from permanent inactivation of COX-1. Given that proplatelets and platelets are not able to resynthesize COX-1 during the 24-hour dosing interval, then the release of newly formed platelets from the bone marrow megakaryocytes (MKs) with unacetylated COX-1, can restore functionally competent level of COX-1 activity. To reproduce these mechanisms, the model includes the following features: i) a compartmental description of aspirin pharmacokinetics (PK), *i*.*e*., its absorption/distribution/metabolism/excretion (ADME); ii) aspirin pharmacodynamics (PD), *i*.*e*., its ability to inactivate COX-1 in proplatelets, platelets and bone marrow MKs; and iii) a detailed description of a turning-over population of MKs, heterogeneous with respect to the maturation stage, and of the population of platelets originating from them [12].

The model was developed by using a combination of “bottom-up” and “top-down” approaches [11]. Briefly, the model structure was built based on the available biological/physiological knowledge, and most parameters were fixed to values inferred from the literature, while only a few key parameters were calibrated based on the available experimental data in healthy subjects and patients off and on low-dose aspirin [13–15]. The model adequately predicted the main features of COX-1 dynamics observed both in healthy subjects and in clinical conditions characterized by an increased platelet turnover [11] or reduced aspirin bioavailability [13].

The first aim of the present study was a model-assessment of the role of platelet progenitors as a hidden drug target, which was often suggested but never actually measured [16–18] since bone marrow MKs are not accessible for repeated daily sampling and measurements of COX-1 activity and inhibition in healthy subjects. To this end, *in silico* experiments were designed both at the cellular and whole-body level and a sensitivity analysis was performed to quantify the role of aspirin PK and PD and of platelets and MK turnover in determining the extent and duration of COX-1 inhibition by a standard regimen of low-dose aspirin administered once daily.

The second aim was to explore the use of the model to design precision aspirin dosing regimens in patients with accelerated platelet turnover or clinical conditions hypothesized to be associated with possible reduction in aspirin bioavailability. *In silico* experiments were performed at whole body level, and the antiplatelet effect was predicted over a wide range of aspirin dosing strategies.

## Materials and methods

### The aspirin PB-PK/PD model

**Fig 1:**
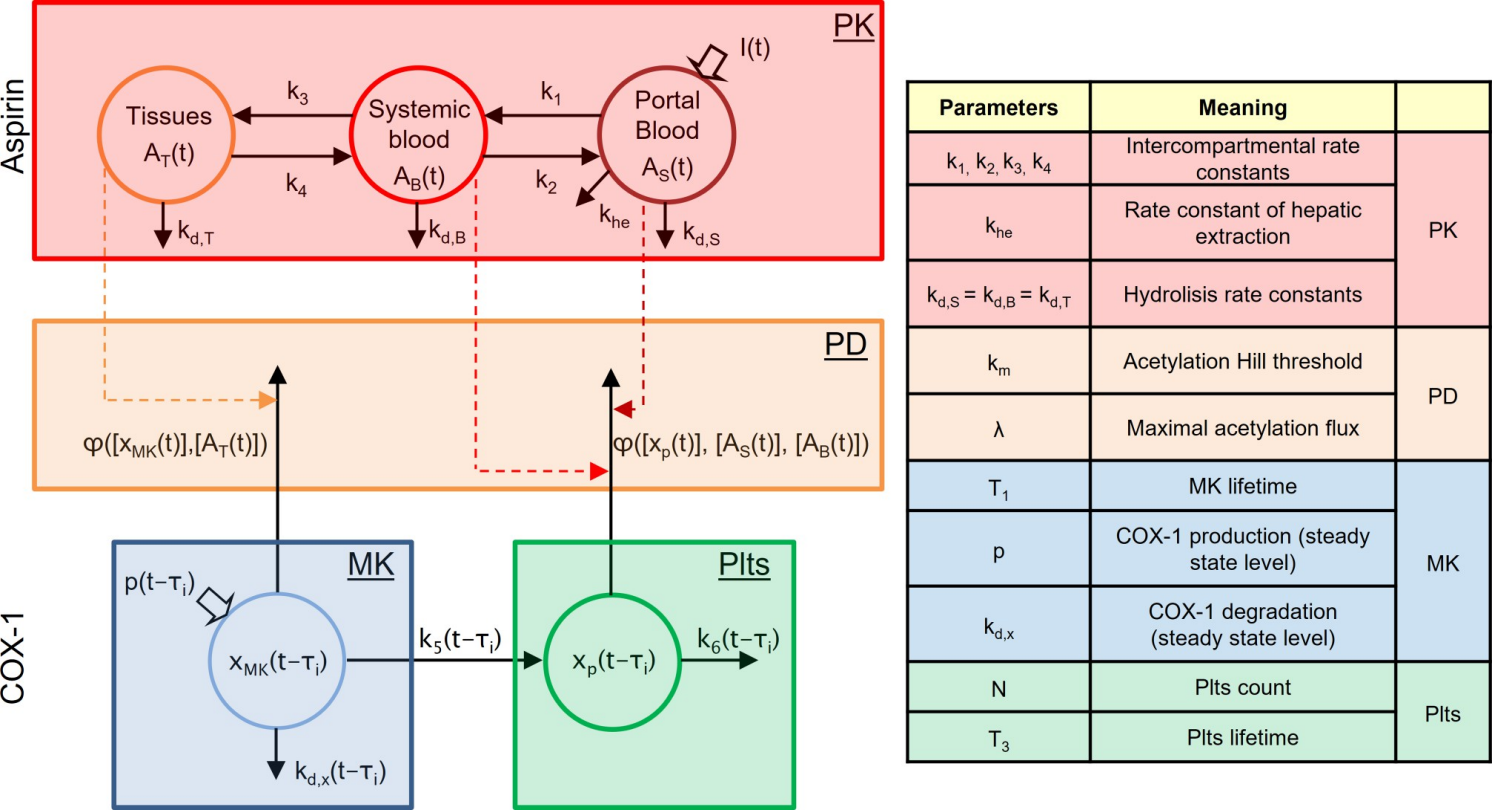
Model of aspirin. The model of aspirin–cyclooxygenase (COX-1) interaction in a single megakaryocyte (MK)-platelet unit. (Left–top panel) the linear three compartment model accounting for whole body aspirin pharmacokinetics. A_T_(t), A_B_(t), A_S_(t) represent aspirin quantity in tissues, systemic blood and portal blood, respectively; I(t) is the model input accounting for the aspirin dose and its absorption by the gastrointestinal tract; k_1_, k_2_, k_3_, k_4_ are the exchange rate constants, k_d,T_, k_d,B_, k_d,S_ are hydrolysis rate constants, k_he_ denotes hepatic extraction; arrows indicate fluxes of material among compartments, dashed arrows controls. (Left–middle panel) COX-1 acetylation flux φ induced by aspirin, expressed as a Hill (sigmoidal) function of COX-1 and aspirin concentrations. (Left–bottom panel) The non-linear two-compartment model of unacetylated COX-1 dynamics in a single unit. x_MK_(t- τ_i_) and x_P_(t- τ_i_) represent COX-1 in the unit, i.e. in a number of MK cells born at time τ_i_ and in the platelets originating from them; p(t-τ_i_) is COX-1 production; k_d,x_(t-τ_i_), k_5_(t-τ_i_), k_6_(t-τ_i_) are the rate constants of: degradation inside the MKs, transfer from MKs to platelets, disappearance due to peripheral platelet destruction, respectively. *Right*: model parameters used in the sensitivity analysis and their meaning.

The PB model of the antiplatelet effect of low-dose aspirin that we previously described [11] considers a population of MKs and platelets originated from them, heterogeneous with respect to differentiation, maturation and proliferation stages, according to the most recent evidence on human megakaryopoiesis [12]. Briefly, the model considered that at each time “*t”*, there are some immature MKs committed from stem cell hematopoietic progenitors, others that are in the maturation stage, others that are in the “proliferation stage”, *i*.*e*., mature MKs actively generating platelets, depending on the lag between “*t”* and the time “*τ”* at which each MK started the maturation process. During proliferation, each MK generates a number of platelets, which remains constant during platelet lifetime and then decreases to zero during the apoptotic phase. Model equations, summarized in the supporting information, account for the presence of *n* MK-platelet units, each consisting of a number of MK cells born at a similar time *τ*_*i*_ and of platelets originating from them. Different units share the above dynamic mechanisms, but their time courses are shifted, according to the time *τ*_*i*_ of MK birth. At a single unit level, aspirin-COX-1 interaction is modeled as shown in **Fig 1**. The model includes: i) a nonlinear two-compartment model, representing COX-1 turnover in the MK unit born at time *τ*_*i*_ (MK submodel) and in the platelets (platelet submodel) originating from it; ii) acetylation flux, φ(t), accounting for the irreversible inactivation of COX-1 by aspirin in MKs and platelets (PD submodel); and iii) a linear three-compartment model of aspirin kinetics (PK submodel), common to all MK-platelet units, able to predict the time course of aspirin concentration in MKs (tissue compartment) and circulating platelets (portal and systemic blood), obtained by summing up the contributions of all circulating platelets. The most relevant model parameters are listed in **Fig 1** and their values are shown in the supporting information: rate constants of aspirin PK, parameters of the sigmoidal function used to model COX-1 acetylation by aspirin, platelet half-life and count; MK lifetime and steady state values of COX-1 production and fractional turnover, after the burst in transcription during the initial stage of MK maturation.

**Fig 2:**
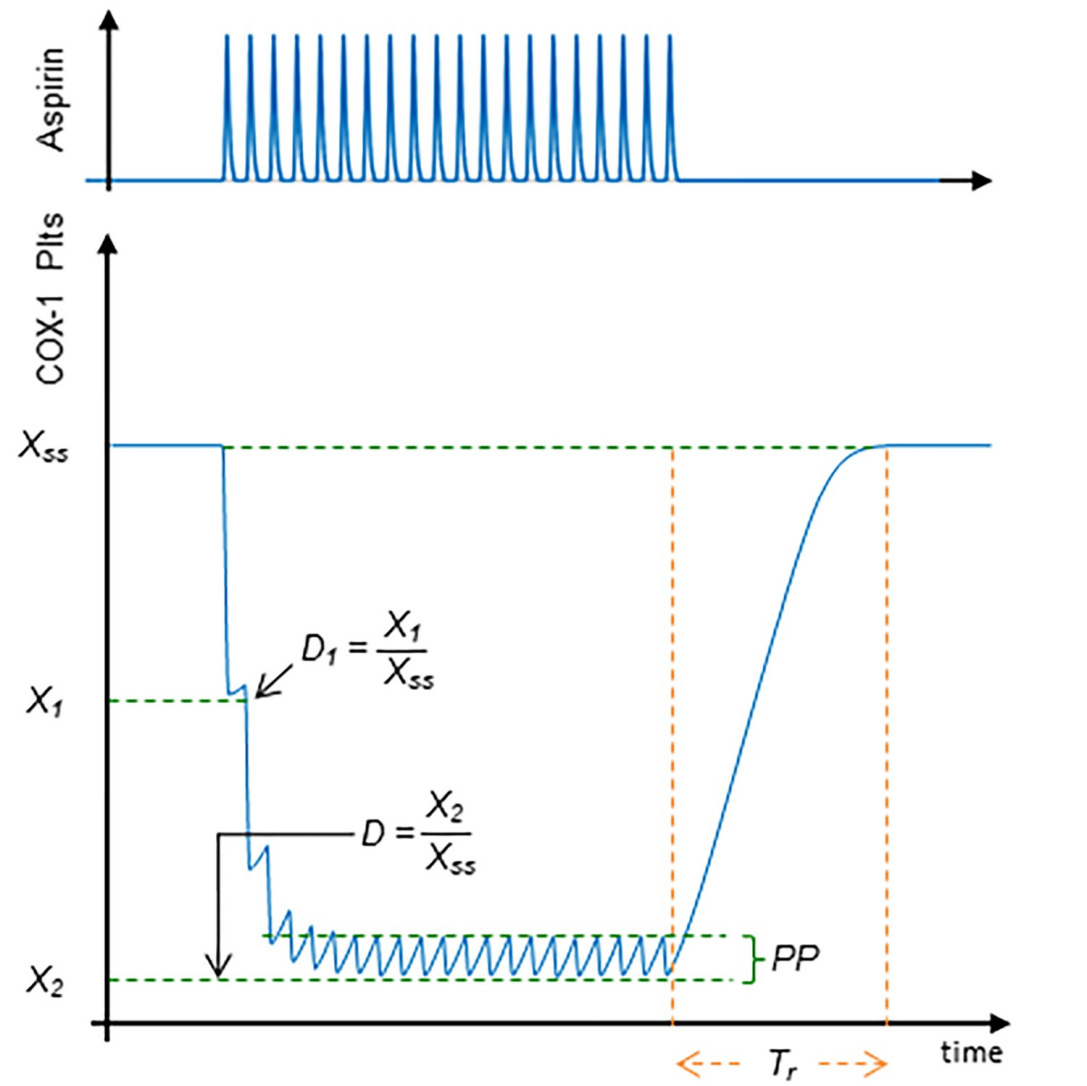
Features of COX-1 during daily aspirin administration. Model-predicted cyclooxygenase (COX-1) in circulating platelets during 3 weeks of low-dose daily aspirin administration. The aspirin concentration in systemic blood (top panel) and unacetylated COX-1 (bottom panel). Steady-state value (X_ss_), decline after the first aspirin dose expressed both in absolute terms (X_1_) and normalized to the steady-state value (D_1_), overall reduction, expressed both in absolute terms (X_2_) and normalized to the steady-state value (D), peak-to-peak interval (PP) and recovery time (T_r_) are also shown.

For simulations at whole body level, the model output is the total amount of unacetylated, enzymatically active, COX-1 (from here on the term COX-1 refers to the unacetylated form) in circulating platelets. **Fig 2** shows the predicted pattern before, during and after a daily low-dose aspirin administration for three weeks in healthy subjects.

### Sensitivity analysis

Due to model complexity, we adopted the classical definition of sensitivity [19], using first-order partial derivatives to express the effect of a single parameter perturbation on the variable of interest, in the present case being the model output shown in **Fig 2**, denoted by X

$${\tilde{S}}_{i}(t,\gamma )=\frac{\partial X(t;p)}{\partial p_{i}(\gamma )}$$ (1)

where ${\tilde{S}}_{i}(t,\gamma )$ is the sensitivity of X at time *t* with respect of a perturbation of the p_i_ parameter at time γ and **p** = [p_1_,…,p_N_] is the vector containing the model parameters. The perturbation can be applied at any time, but it is usually set at the initial time γ = 0 and for this reason the term γ is usually dropped out. For the sake of comparison, the sensitivities are usually normalized as follows:

$$S_{i}(t)=\frac{\partial X(t;p)}{\partial p_{i}}\frac{p_{i}}{X(t)}$$ (2)

Being the sensitivity a function of time, sensitivity analysis allows one to measure both the extent to which a single parameter affects the system behavior and when it happens. However, in order to facilitate the ranking of model parameters based on their influence on model output, it is convenient to define some indices. Consolidated metrics are:

1. mean sensitivity over the entire simulation interval, obtained by averaging N samples taken at times t_k_:
$$S_{i}^{M}=\frac{1}{N}\sum_{k=1}^{N}S_{i}(t_{k})$$ (3)
2. sensitivity based on the Fisher information matrix [20]:
$$S_{i}^{F}=\frac{1}{N}\sum_{k=1}^{N}S_{i}^{2}(t_{k})$$ (4)
3. sensitivity based on infinite norm [20]:
$$S_{i}^{N}=\underset{k}{\max}\left(\left|S_{i}(t_{k})\right|\right)$$ (5)

Additional quantitative indices were defined able to reflect the influence of a single parameter on the main features of the COX-1 pattern shown in **Fig 2**, namely:

■ the plateau value X_ss_, related to the steady state present before aspirin administration and restored a few days after aspirin withdrawal;
■ the inhibition by the first aspirin dose, D_1_, defined as the minimum value X_1_ reached following the first dose normalized to the plateau value, namely D_1_ = X_1_/ X_ss_;
■ the overall inhibition D, defined as the nadir value C_2_ reached after a few aspirin administrations normalized to the plateau value, namely D = X_2_/ X_ss_;
■ the peak-to-peak value, PP, of oscillations due to the daily aspirin administration;
■ the recovery time, T_r_, after aspirin withdrawal, as the time needed to restore the plateau value (within 3%).

Let’s call $F\in \{X_{ss},D_{1},D,PP,T_{r}\}$ the generic feature extracted from X(t) with no parameter perturbation and F+ΔF the generic feature extracted from X(t) after the perturbation Δp_i_ of the parameter p_i_. Then, following the definition of normalized sensitivity (Eq 2), feature-based sensitivity indices were defined to measure the relative effect on each feature of a perturbation of a model parameter p_i_:

$$S_{F,i}=\frac{\Delta F}{\Delta p_{i}}\frac{p_{i}}{F}$$ (6)

All sensitivities were evaluated with respect to the 13 parameters listed in **Fig 1**, thus 6x13 = 78 sensitivity indices were calculated, 13 related to the sensitivity of COX-1 pattern over the entire simulation interval and 13 related to each of the five features.

### Implementation

The model consists of two ordinary differential equations for each MK-platelet unit, plus the three differential equations describing aspirin PK. To account for the heterogeneity of units with respect to the MK maturation stage, 5000 units were simulated, each representative of a set of cells born at a similar time.

To characterize the model behavior, unacetylated COX-1 profile in MKs and platelets, expressed as % of the total (unacetylated and acetylated) COX-1, were simulated under a variety of aspirin regimens (single or repeated administrations) and doses (from 25 to 200 mg), both in single MK-platelet units and at whole body level. Parameters were first fixed to values previously reported [11], inferred from the literature and/or calibrated using measurements of serum thromboxane B_2_ (sTXB_2_) as proxy of COX-1 activity in peripheral platelets of 17 healthy subjects and with physiological hematopoiesis.

Then, to exemplify the use of the model as a tool to personalize antiplatelet therapy, simulations at whole body level were also performed by fixing model parameters to values previously reported in two different conditions, characterized by:

- accelerated platelet turnover, as in essential thrombocythemia (ET) [11, 21].
- reduced aspirin bioavailability, as in obesity [13].

The complex structure of the model forced the use of the indirect method, as reported in [19], to implement the sensitivities (Eq 2), which makes use of the finite difference approximation:

$$S_{i}(t)=\frac{X(t;p_{1},\ldots ,p_{i}+\Delta p_{i},\ldots ,p_{N})-X(t;p_{1},\ldots ,p_{i},\ldots ,p_{N})}{\Delta p_{i}}\frac{p_{i}}{X(t)}$$ (7)

where Δpi is the parameter perturbation. A 1% parameter increment was considered, that is Δp_i_/p_i_ = 0.01.

All calculations were performed in MATLAB (Math Works, Natick, MA), a numerical computing environment and programming language used in science and engineering, by extending the software we originally developed to implement our model [11] so as to include sensitivity analysis.

## Results

### Model behaviour under healthy conditions

**Fig 3:**
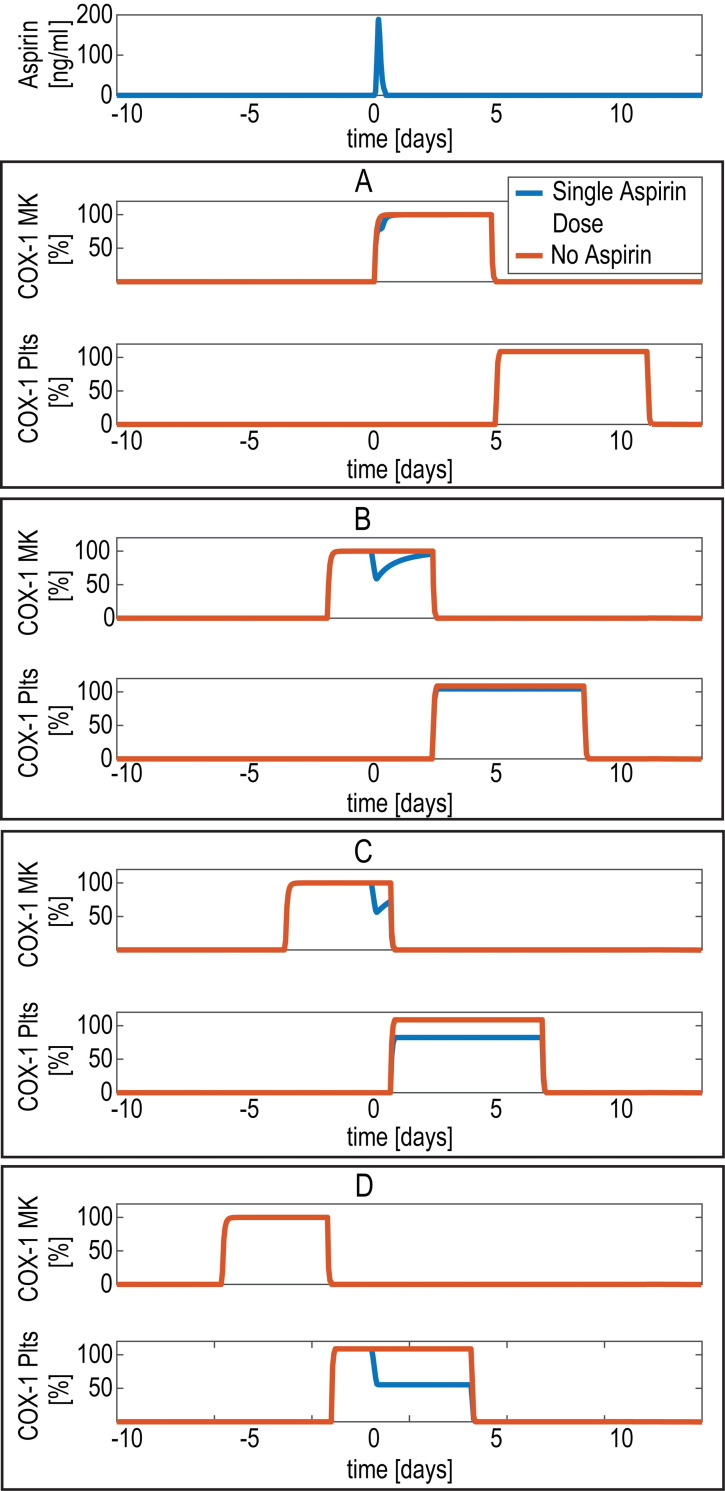
Model-predicted COX-1 during a single aspirin dose in a single (MK)-platelet unit. Model-predicted cyclooxygenase (COX-1) in a single megakaryocyte (MK)-platelet unit during a single low-dose aspirin administration in healthy conditions. The aspirin concentration in systemic blood (top panel); unacetylated COX-1 in MKs and platelets, both expressed as % of total COX-1, for four different units, born at different times (panels A-D).

The time course of unacetylated COX-1 (referred to as COX-1) in MKs and platelets following a single low-dose (*i*.*e*., 100 mg) of aspirin administration is shown in **Fig 3** for four different MK-platelet units, characterized by four different times of birth, so that the time of aspirin administration is shortly after the time of MK birth (panel A), during MK maturation (panel B), close to the end of MK maturation (panel C) and during platelet lifetime (panel D). In newly born MKs (panel A), the effect of a single aspirin dose is transient and minimally impacts on platelets, since new COX-1 expressed in bone marrow progenitors compensates for the loss of enzymatic activity through the permanent acetylation. Similarly, in MKs during the maturation stage (panel B), COX-1 inhibition is compensated by new COX-1 generation. In MKs close to the end of the maturation stage (panel C), the compensation is only partial and less COX-1 is transferred to platelets. Aspirin effect is permanent in platelets (panel D) due to their inability to synthetize COX-1 *de novo*.

**Fig 4:**
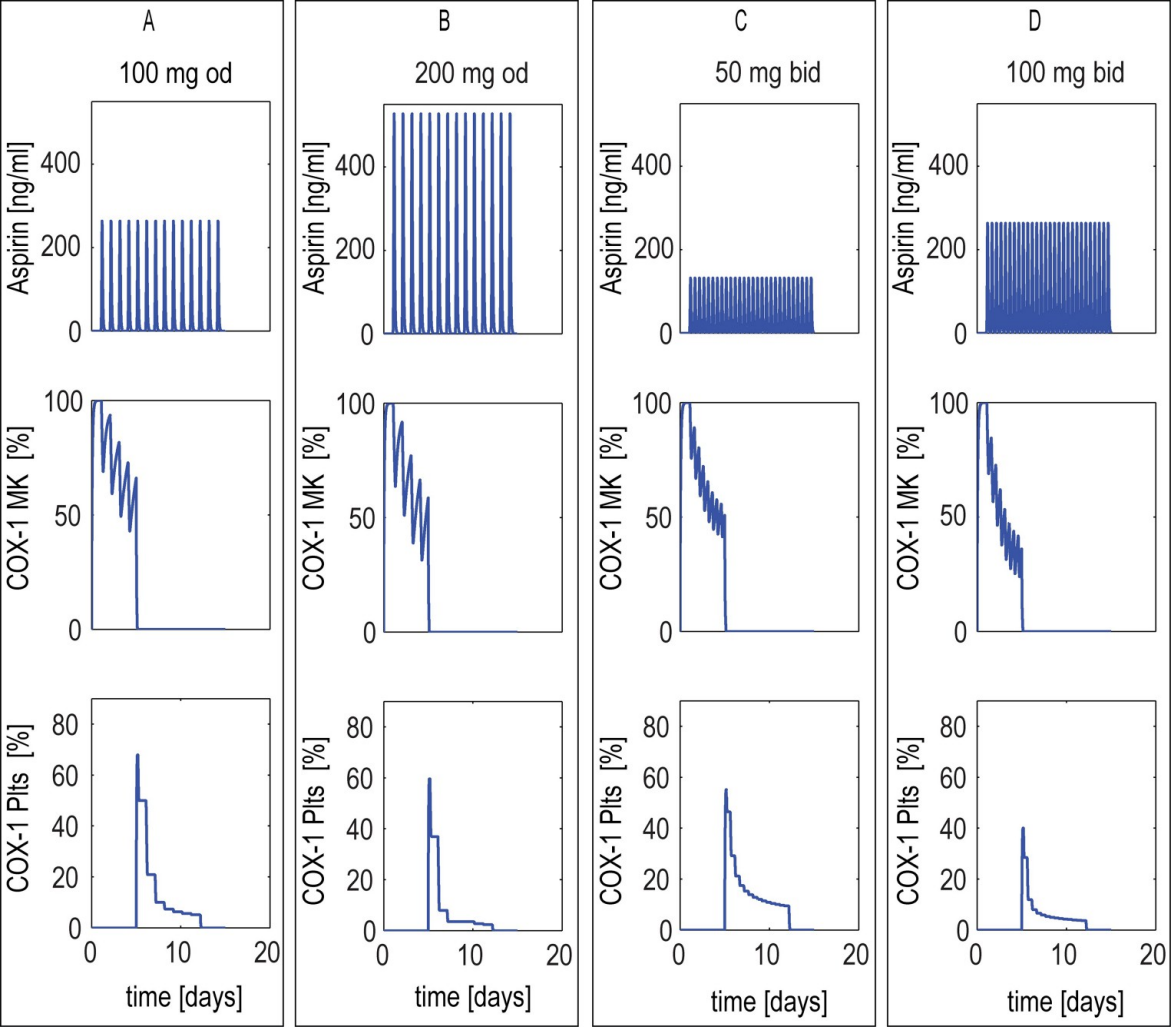
Model-predicted COX-1 during a single aspirin doses in a single (MK)-platelet unit. Model-predicted cyclooxygenase (COX-1) in a single megakaryocyte (MK)-platelet unit during its entire lifespan and during once-(od) or twice-(bid) daily aspirin administration in healthy conditions. The aspirin concentration in systemic blood (top panel); unacetylated COX-1 in MKs (middle panel) and platelets (bottom panel), both expressed as % of the total COX-1 inside the unit, during aspirin administration of: 100 or 200 mg od (panels A, B) and 50 or 100 mg bid (panels C, D).

When the once-daily (od) aspirin dosing is repeated once daily (od), COX-1 activity is partially reduced both in MKs to ~63% of the initial activity at the end of their life) and in platelets (from ~64% it falls down to ~7% at the end of their life) (**Fig 4** - panel A). Doubling the aspirin dose once-daily resulted in marginal differences in inhibition with respect to the standard dose (**Fig 4** - panel B) both in MK COX-1 (~54% at the end of their life) and platelets (from ~54% down to ~3%). On the other hand, a twice daily (bid)low-dose aspirin administration of either 50 mg or 100 mg can limit the amplitude of COX-1 activity oscillations during the 24 hour dosing interval (**Fig 4** - panels C and D), with lower values of COX-1 activity in MKs at the end of their life (to ~54% and 36%, respectively), lower in platelets at the beginning of their life (~58% and 40%, respectively), slightly higher at the end (~13% and 5%, respectively).

**Fig 5:**
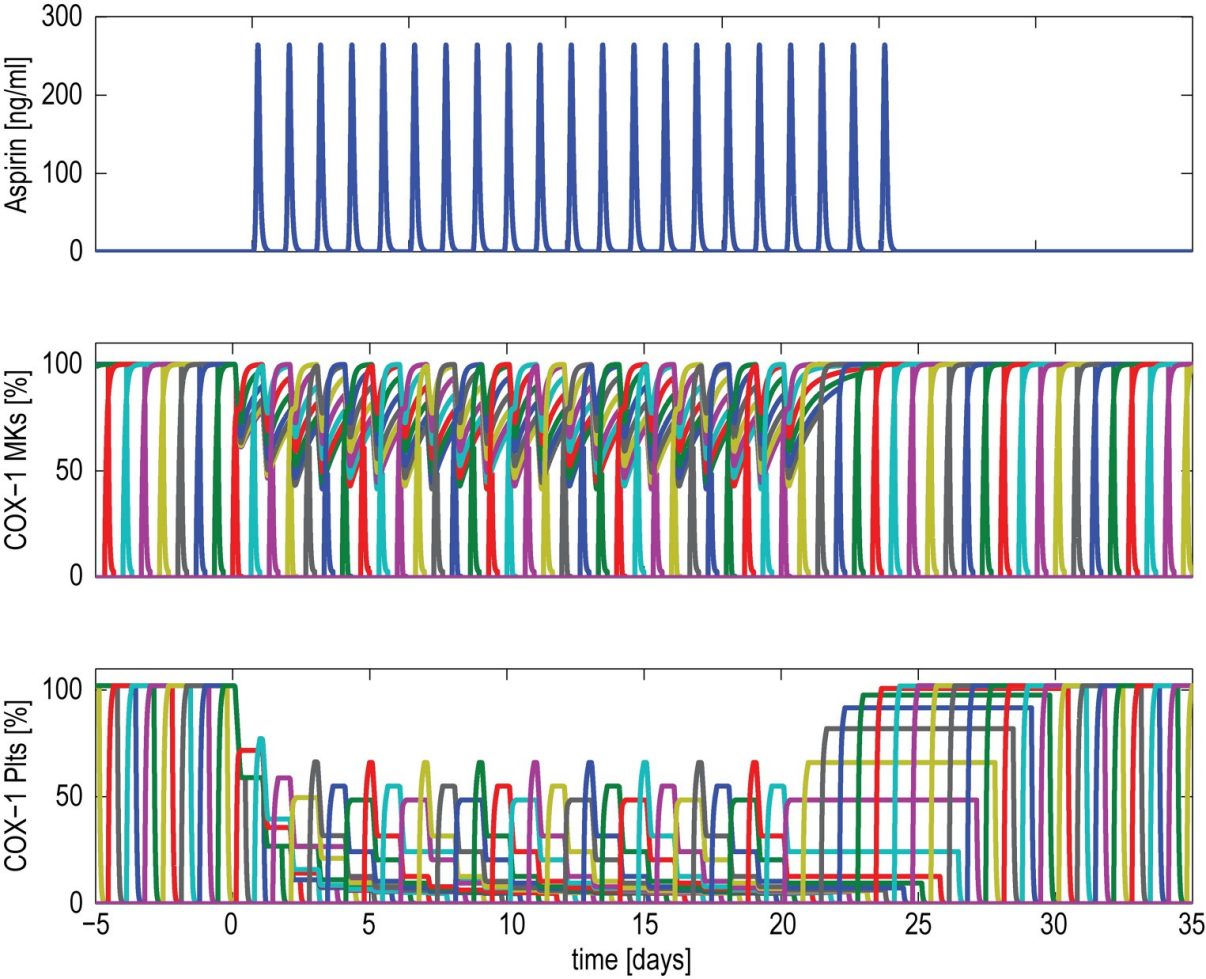
Model-predicted COX-1 in multiple (MK)-platelet units. Model-predicted cyclooxygenase (COX-1) in multiple megakaryocyte (MK)-platelet units during low-dose daily aspirin administration in healthy conditions. The aspirin concentration in systemic blood (top panel); unacetylated COX-1 in MKs (middle panel) and platelets (bottom panel) of multiple units, both expressed as % of the total COX-1. Different colours are representative of different MK-platelet units.

The superposition of COX-1 enzymatic profiles in multiple units, before, during and after a three-week once daily low-dose aspirin administration is shown in **Fig 5**.

**Fig 6:**
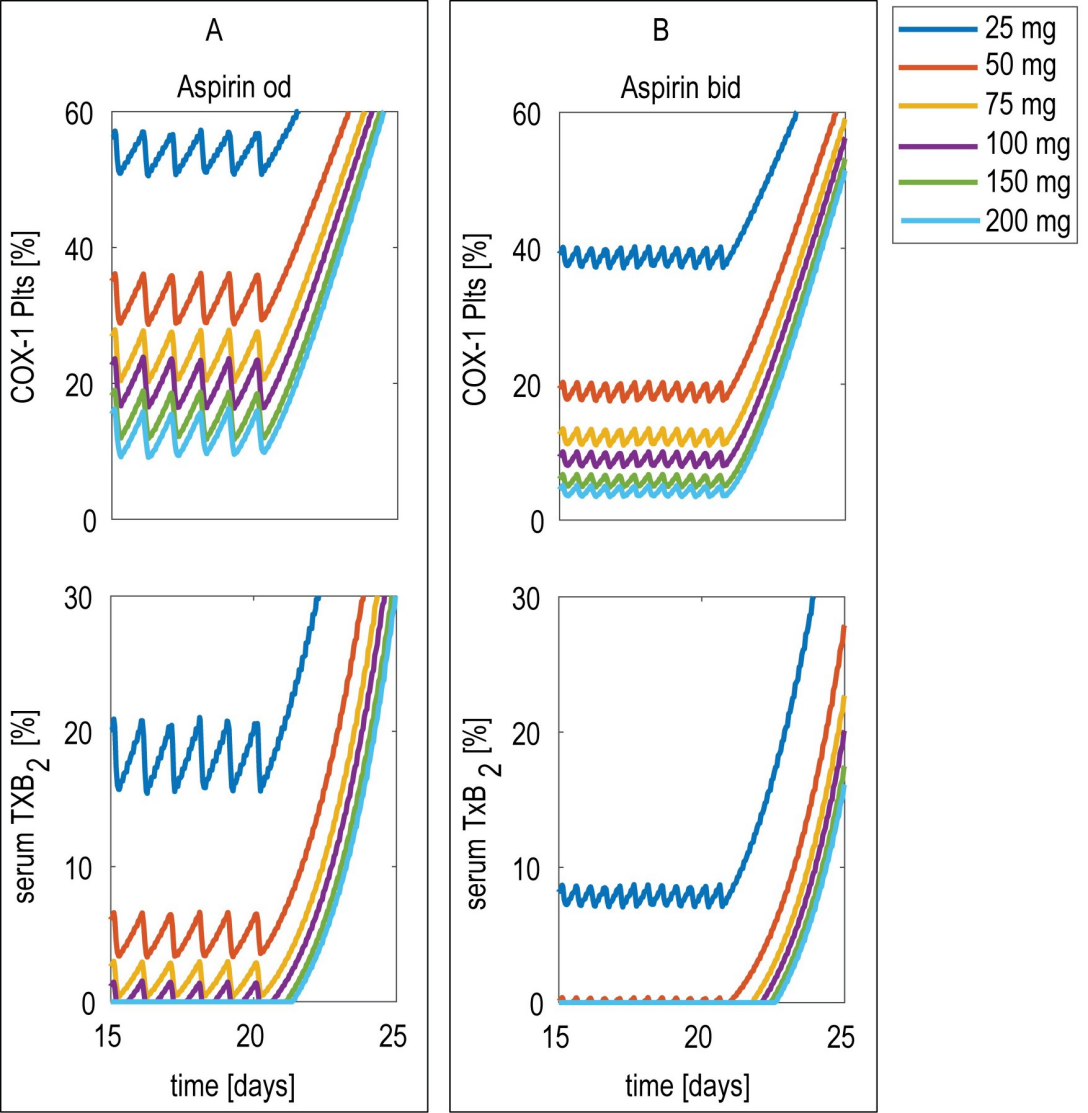
Model-predicted whole body COX-1 in healthy subjects. Model-predicted whole body cyclooxygenase (COX-1) and its conversion to serum thromboxane B_2_ (sTXB_2_) during once-(od) or twice-(bid) daily aspirin administration in healthy condition. Unacetylated COX-1 in circulating platelets, expressed as % of the total COX-1 (top panel) and sTXB_2_ as proxy of COX-1 activity (bottom panel), during od (panel A) and bid (panel B) administration of different aspirin doses.

The model was then used to predict the COX-1 activity patterns in the systemic in circulating platelets and the level of residual serum TXB_2_ (**Fig 6**) with different aspirin doses administered once (panel A) or twice (panel B) daily.

sTXB_2_ reflects platelet COX-1 maximal enzymatic activity [22], and is a validated biomarker of aspirin PD [23]. In keeping with the simulations shown in **Fig 4** for an individual unit, even at systemic level the amplitude of COX-1 activity daily oscillations is reduced with a low-dose, bid dosing, and COX-1 inhibition is steadier. However, apart from the lowest dose, the conversion of COX-1 to sTXB_2_ does not allow to monitor any difference in the maximal suppression, since a threshold is present in the conversion curve, reflecting the fact that virtually no sTXB_2_ is generated if unacetylated COX-1, expressed as percentage of total COX-1, is below ~20% [24].

### Model-informed precision dosing of low-dose aspirin in conditions of high platelet turnover

**Fig 7:**
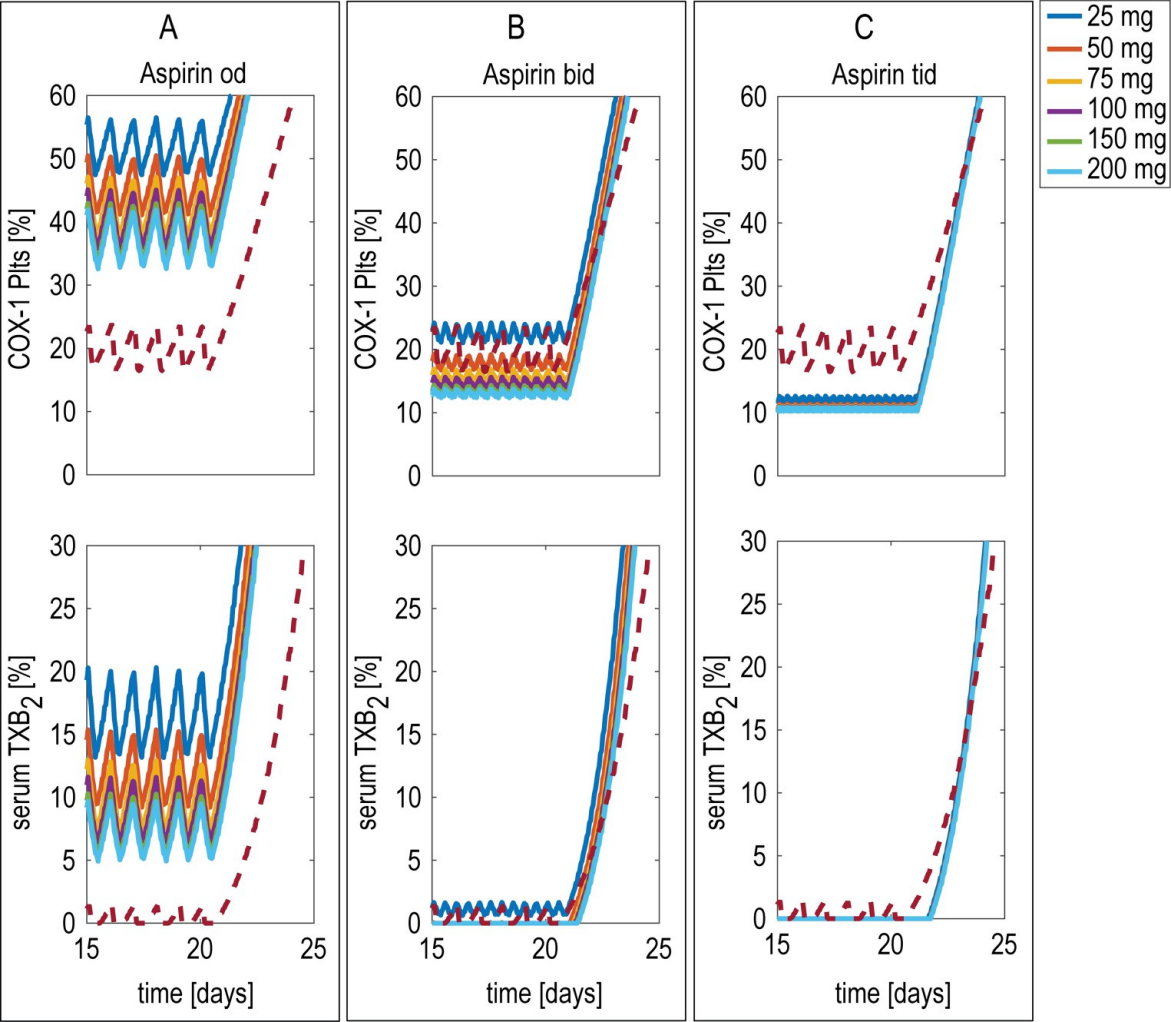
Model-predicted whole body COX-1 in essential thrombocythemia. Model-predicted whole body cyclooxygenase (COX-1) and its conversion to serum thromboxane B_2_ (sTXB_2_) during once-(od) or twice-(bid) or three times-(tid) daily aspirin administration in essential thrombocythemic patients. Unacetylated COX-1 in circulating platelets, expressed as % of the total COX-1 (top panel) and sTXB_2_ as proxy of COX-1 activity (bottom panel), during od (panel A), bid (panel B) and tid (panel C) administration of different aspirin doses. Dashed line represent healthy subjects treated with 100 mg od.

In our previous study [11], model parameters (reported in S1 Table in S1 File, as well as in our previous study [11]) were adjusted to reproduce the experimental measurements of sTXB_2_ production by blood platelets of 24 patients with ET, which is an extreme paradigm of accelerated platelet turnover [25]. In order to investigate the effects of different low-dose aspirin regimens in patients characterized by this condition, *in silico* experiments were performed with different aspirin doses administered once-, twice- or three-times daily. The results of these simulations are shown in **Fig 7** and indicate that not even the highest dose if administered once-daily can suppress COX-1 activity in ET to values close to those observed in healthy subjects treated with the standard daily regimen (eg 100 mg). Similar levels are reached either with a 50 mg bid or 25 mg tid dosing regimen, thus showing a clear biochemical superiority of multiple daily dosing as compared to the standard once daily regimen in conditions associate with an accelerated renewal of the drug target.

### Model-informed precision dosing of low-dose aspirin in conditions of reduced aspirin bioavailability

**Fig 8:**
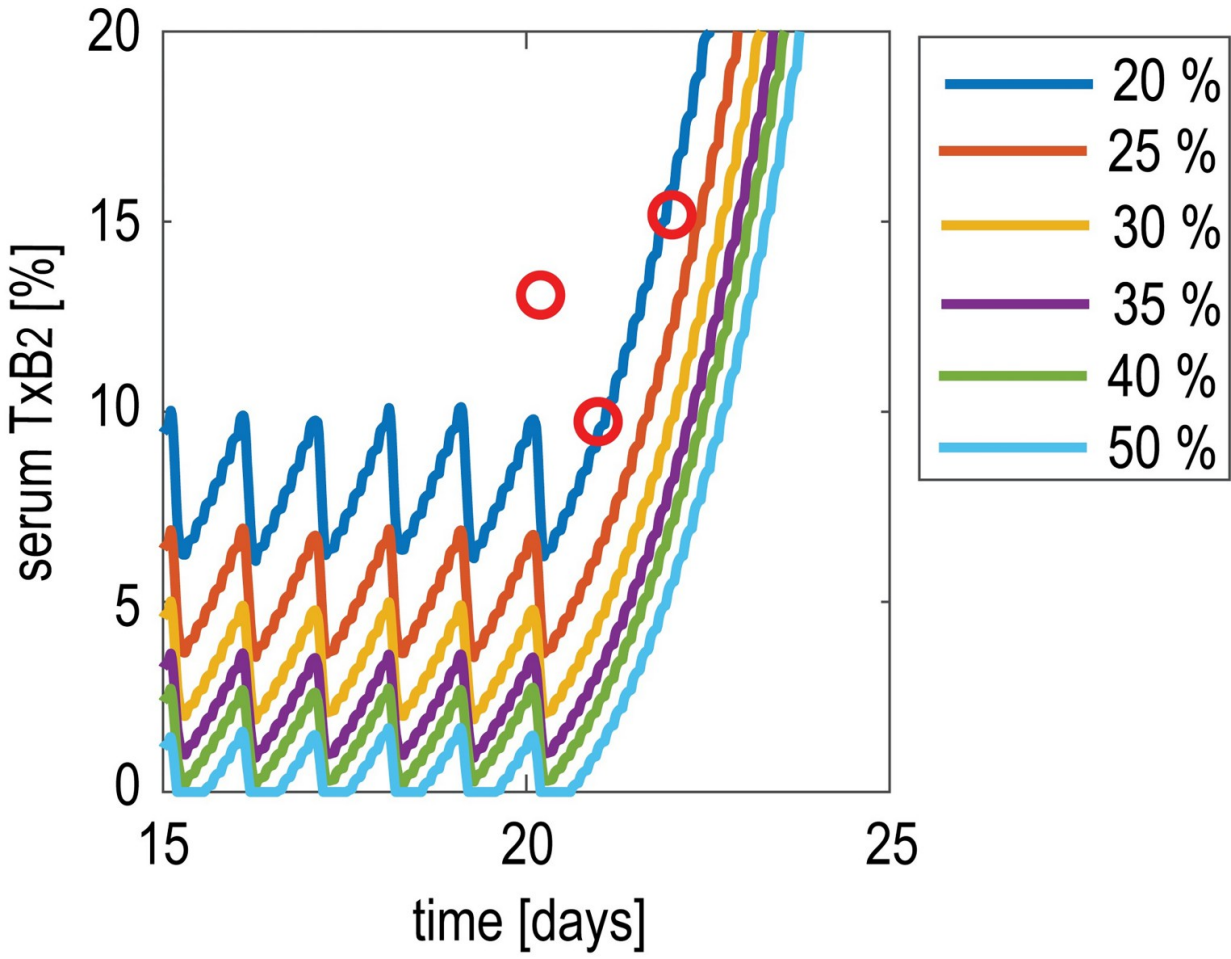
Model-based inference of aspirin bioavailability in a severely obese subject. Comparison between model predictions of sTXB_2_ profiles for different values of bioavailability (lines) and data measured in an obese subject (red dots) suggests for this subject a bioavailability as low as 20%, which is the value best able to reproduce the data.

As a second case study, we considered the effect of increasing body size, and specifically of obesity, defined as body mass index [BMI] ≥30 kg/m^2^, which has been associated with reduced aspirin responsiveness [13, 26] likely explained by a reduced systemic bioavailability of the drug, likely due to increased hydrolysis by esterares in the intestinal fat [26, 27]. Thus, the aspirin dose was increased in obese subjects according to its systemic bioavailability rate and higher volume of distribution secondary to obesity-associated changes [28]. The model was then used to design the optimal dose in obese subjects, starting from a few experimental measurements of sTXB_2_ following a standard low-dose aspirin regimen [13]. **Fig 8** illustrates the case of a representative obese subject: sTXB_2_ data are superimposed to a set of model-predicted sTXB_2_ time courses, simulated by assuming different levels of drug bioavailability. Comparison between experimental data and model predictions suggests that for BMI associated with severe obesity (i.e., 46.7 kg/m^2^) aspirin bioavailability is decreased down to 20%, since this value is best able to fit the experimental sTXB_2_ data.

### Sensitivity analysis

**Fig 9:**
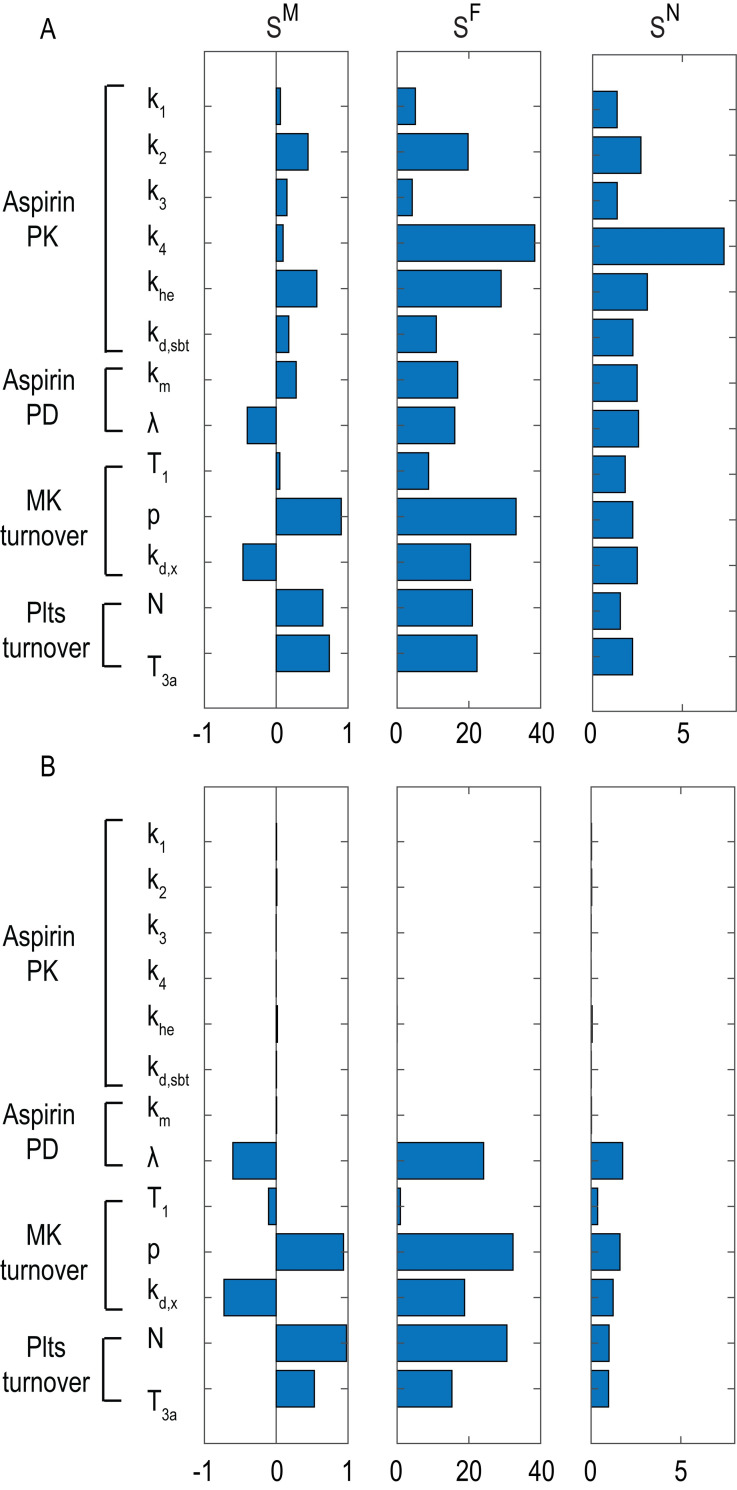
Overall sensitivity analysis. Effect of a perturbation in a model parameter (specified on the left side) on overall COX-1 pattern in terms of mean (1^st^ column), Fisher-based (2^nd^ column) and infinity norm-based (*3*^*rd*^ *column*) sensitivities. Sensitivities are normalized as indicated in Eq 2, i.e. a bar equal to 1 indicates that a 1% increase of the model parameter specified on the left side reflects in an 1% increase of the mean (1^st^ column), quadratic mean (2^nd^ column), maximal (3^rd^ column) value of COX-1 pattern.

**Fig 10:**
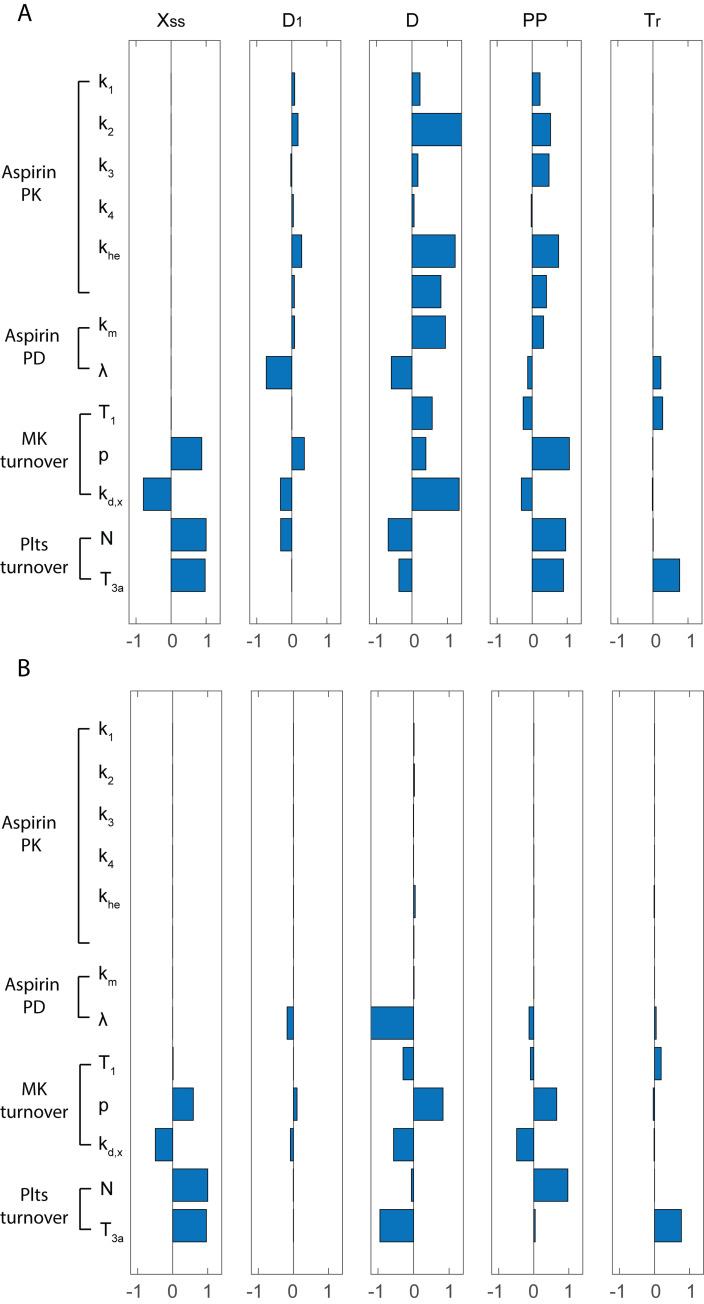
Feature sensitivity analysis. Effect of a perturbation in a model feature, namely the plateau value C_ss_, the inhibition due to the first aspirin dose D_1_, the overall inhibition D, the peak-to-peak value PP, the recovery time T_r_. Panel A refers to healthy subjects, panel B to essential thrombocythemia patients. Sensitivities are normalized as indicated in Eq 6, e.g., a bar equal to -1 in the 1^st^ column indicates that a 1% increase of the model parameter specified on the left side reflects in a 1% decrease of X_ss_.

The sensitivity of the platelet COX-1 pattern to a perturbation of model parameters listed in **Fig 1** were evaluated starting from values representative of physiological (**Figs 9** and **10** - panel A) and pathological, *i*.*e*. ET (**Figs 9** and **10** - panel B), conditions. Only in a few cases the sensitivity values, evaluated in terms of either the mean (**Fig 9**, first column) or feature-based sensitivity (**Fig 10**), exceed the +1/-1 range, indicating that a 1% perturbation in a single model parameter rarely induces an amplified effect on the mean COX-1 pattern as well as on its main features (defined in the Materials and Methods section and in **Fig 2**). On the other hand, the sensitivity values, evaluated in terms of Fisher matrix or the infinity norm (**Fig 9**), exceed the +1/-1 range for most parameters, suggesting the presence of positive and negative fluctuations during the dynamical evolution of the system and/or amplified effects of parameter perturbation at some specific times.

The sensitivity indexes of the COX-1 pattern (Eqs 3, 4, 5) under physiological conditions (**Fig 9** - panel A) confirm the important role of parameters related to COX-1 turnover in MKs and platelets, *i*.*e*., COX-1 production and utilization in MKs as well as platelet number and lifespan.

As regards to the five parameters shown in **Fig 2**, sensitivity analysis indicates that only those related to COX-1 turnover in MKs and platelets influence the COX-1 plateau level (X_ss_, second column in **Fig 10** - panel A). The COX-1 inhibition following the first aspirin dose (D_1,_ third column in **Fig 10** - panel A) is highly sensitive to aspirin PD, namely to the maximal value of COX-1 acetylation induced by aspirin. Conversely, several parameters influence the two features related to the effect of repeated aspirin dosing, such as the overall COX-1 inhibition (D, fourth column in **Fig 10** - panel A) and the peak-to-peak interval (PP, fifth column in **Fig 10**- panel A). Finally, the recovery time interval after aspirin withdrawal is influenced by the platelet lifespan, and, to a lesser extent, by MK lifespan and acetylation flux, in keeping with the notion that acetylation by aspirin induces a permanent inactivation of COX-1, so that only the release of newly formed platelets with unacetylated COX-1, originating from newly formed MKs, allows to restore the peripheral COX-1 plateau level.

ET is characterized by a marked increase in MK mass and proliferation and consequent increased platelet number [25]. Under this pathological condition, the COX-1 pattern shows a less marked inhibition and a more rapid recovery phase. The model was able to reproduce this condition by means of a decrease in MK lifetime, an increase in COX-1 expression in MKs and an increase in the maximal value of acetylation flux, which is proportional to the platelet count. When the sensitivity analysis was performed in the ET setting (**Figs 9** and **10** - panel B), the major finding is that a 1% perturbation in any aspirin PK parameter is no longer able to induce a visible influence on COX-1 patterns and/or features, due to the prevailing influence of MK and platelet turnover on COX-1 dynamics, thus requiring a more frequent dosing regimen.

## Discussion

In this study, we developed a computational approach *to* characterize the determinants of extent and duration of platelet COX-1 inhibition by aspirin and design precision dosing in patients with accelerated platelet turnover or reduced drug bioavailability.

The core of our computational approach is the PB PK/PD mathematical model, previously published by our group [11], able to reproduce the antiplatelet effect of low-dose aspirin in different physiological and pathological conditions [8, 13]. The novelty of the present paper is the design, performance and interpretation of a set of in silico experiments able to provide a model-assessment of aspirin action in accessible and non accessible compartments, namely not only whole body as in the previous paper but also cells (MKs and platelets), during a wide range of aspirin regimens in different patient populations.

The first aim of this study was the assessment of the role of platelet progenitors as a relevant, albeit routinely anatomically unreachable, drug target. As shown in **Fig 3**, the model addressed the heterogeneity of the COX-1 response to a single low-dose aspirin administration in MKs and platelets, depending on their maturation and proliferation stages. The assumption that aspirin is able to irreversibly inactivate COX-1 not only in peripheral platelets but also in their bone-marrow progenitors, based on its systemic bioavailability [29] is central to reproduce the persistent COX-1 inactivation in platelets, observed during repeated, once-daily dosing (**Fig 4**). Thus a first key finding of our approach is the validation of a major role of MK and platelet turnover on the extent and duration of COX-1 inhibition by aspirin given at low-doses, which is the reference antiplatelet treatment of cardiovascular patients [30].

The second aim was to assess the reliability of our approach as a tool to help designing precision dosing regimens for selected patients. We explored three different conditions: physiological conditions, conditions characterized by accelerated platelet production such as ET, or by reduced systemic drug bioavailability such as severe obesity.

Under physiological conditions, the model predicts that a dose of aspirin as low as 50 mg administered more frequently (bid) can suppress platelet COX-1activity (**Fig 6**) to levels close to those observed with a standard once daily administration of 100 mg, and that similar effects are induced by higher doses (100 mg bid and 200 mg) given once daily. These data are consistent with large trials showing the efficacy of aspirin daily doses up to 100 mg in cardiovascular prevention [31], with no further clinical benefits from increasing once-daily doses [31]. However, in all cases, the model predicts that a virtually complete suppression of platelet COX-1 activity (as reflected by sTXB_2_ measurements) can be also reached by a bid regimen as low as 25 mg. Consistently, the European Stroke Prevention Study 2 investigated a bid aspirin regimen of 25 mg showing a significant benefit vs. placebo in preventing recurrent cerebrovascular events [32], and a pharmacodynamic study on patients with acute coronary syndrome showed the capacity of a 20 mg bid dosing in fully inhibition COX-1 dependent platelet function [33].

While the aspirin regimen is well established in subjects with a physiological platelet turnover and normal-to-overweight body size, this is uncertain under conditions characterized by high platelet production, such as ET [34]. Model predictions obtained by using parameter values (reported in S1 Table in S1 File, as well as in our previous study [11]) adjusted to simulate the accelerated platelet turnover associated with ET (**Fig 7**) suggest that only multiple daily dosing regimens can achieve sTXB_2_ levels similar to the ones of healthy subjects. Thus, our PB-PK/PD analysis supports the notion that multiple (either bid or tid) daily regimens are superior with respect to the traditional once daily regimen under conditions of accelerated platelet turnover. Indeed, the ARES phase II trial has recently showed a superior efficacy of multiple (both bid and tid) daily low-dose aspirin regimens in adequately suppressing sTXB_2_, as compared to the standard od regimen [31]. Moreover, the same trial showed that a tid regimen was similar to a bid regimen in the degree of platelet COX-1 inhibition [31, 35, 36] which is consistent with our in silico prediction (**Fig 7**).

Obesity is associated with several metabolic changes, which may potentially affect aspirin systemic bioavailability, in particular processes related to aspirin inactivation before, during and after the liver first pass [26]. Obese subjects (especially with moderate-to-severe obesity, *i*.*e*., with BMI≥35 kg/m^2^) are largely under-represented in cardiovascular clinical trials, or even excluded if severely obese, thus pharmacological and clinical data in these patients are needed, also due to their high cardiovascular risk [6]. To simulate this condition, our PB-PK/PD model was adapted by modifying the parameter related to drug bioavailability, and then used to suggest the precision dose in a severely obese patient, (**Fig 8**). A personalized approach in a cohort of otherwise healthy severely obese subjects, i.e., with an average BMI of 39 kg/m^2^, showed that body size, expressed as both BMI and body weight, influences aspirin pharmacology with a significant inverse relationship [16, 37] so that doubling the once daily dose or giving the same dose bid would restore an adequate suppression of COX-1 activity [13, 38, 39].

Summing up the consistency of our model-based findings with experimental data from large trials in healthy subjects and ET patients supports the potentiality of our approach for designing precision dosing regimens and *in silico* trials. Key findings regard the superiority of reducing the dosing interval *vs* increasing the once-daily dose in conditions of increased platelet turnover, and the model-suggested dose adjustments in conditions of reduced drug bioavailability.

Our PB-PK/PD model was also able to describe the pharmacology of aspirin in both physiological and pathological conditions by modifying specific model parameters (reported in S1 Table in S1 File, as well as in our previous study [11]) that are likely to be associated with the condition under study. A more comprehensive representation of the influence exerted by model parameters on drug response is provided by sensitivity analysis, able to characterize the relative role of individual processes like aspirin PK, PD, MK and platelet turnover, in determining the extent and duration of peripheral platelet COX-1 inhibition by aspirin. Due to model complexity, we adopted the classical definition of sensitivity [19], that investigates the effect of a persistent variation in a single parameter while other parameters are kept constant at their nominal values, and implemented it by finite difference approximation. Other sensitivity approaches are available, e.g., global sensitivity, which considers variations of all input variables simultaneously [40]. Similarly, other implementation methods are available, e.g., based on differentiating the model equations [19]. They represent interesting alternatives to the approach we adopted, but their application to our model is not computationally feasible.

For sensitivity analysis, we selected the 13 most representative parameters (reported in S1 Table in S1 File, as well as in our previous study [11]) and quantified their influence on the COX-1 activity pattern observed before, during and after repeated daily low-dose aspirin administration for three weeks. Under physiological conditions (**Figs 9** and **10** –panel A), our results confirm the important role of the parameters related to thrombopoiesis. The influence of parameters of aspirin kinetics, which is fast (minutes) compared to MK and platelet turnover (days), is limited to those parameters most related to aspirin clearance, and has minor impact in ET (**Figs 9** and **10** –panel B), *i*.*e*., under conditions of accelerated platelet turnover. Under this condition, platelet number, in addition to increased turnover, reduces aspirin responsiveness and a bid regimen appears superior to the traditional once daily regimen.

## Conclusions

*In silico* experiments and sensitivity analyses confirmed that the antiplatelet effect of low-dose aspirin is significantly modulated not only by the conventional PK/PD parameters, but also by the turnover rate of its two distinct cellular targets, namely blood platelets and bone-marrow MKs. Our model, including the two common components of physiologically based PK/PD models, namely drug-specific information (aspirin PK/PD) and biological/physiological information on system properties that are independent of the drug (platelet and MK turnover), appears to be adequate to largely elucidate the dynamic features of low-dose aspirin pharmacology. This model is potentially useful to instruct precision pharmacology, to both design and test personalized drug regimens, as well as to inform decision algorithms in special patient’s populations characterized by aspirin PK alterations (e.g., obese patients) and rare diseases characterized by alteration of platelet and MKs turnover (e.g., essential thrombocythemia) where trials are practically unfeasible and *in silico* trails can be central to the optimal antithrombotic management of those patients.

## Supporting information

S1 File Mathematical model description. (DOCX)
