# Supplementary material for: Physiologically based modelling of the antiplatelet effect of aspirin: A tool to characterize drug responsiveness and inform precision dosing
Source: PLoS One. 2022 Aug 17;17(8):e0268905. doi: 10.1371/journal.pone.0268905 (PMC9385056; doi:10.1371/journal.pone.0268905)
Supplement: S1 File — (DOCX) [file pone.0268905.s001.docx]

**Supporting Information – Mathematical model description**

**Physiologically based modelling of the antiplatelet effect of aspirin: a tool to characterize drug responsiveness and inform precision dosing**

Alberto Giaretta^1,2*^, Giovanna Petrucci^3^, Bianca Rocca^3^, Gianna Maria Toffolo^1^

^1^Department of Information Engineering, University of Padova, Padova, Italy

^2^Department of Pathology, University of Cambridge, Cambridge, United Kingdom

^3^Department of Pharmacology, Catholic University School of Medicine, Rome, Italy.

Here we condense the major mathematical details of the aspirin model shown in figure 1, consisting of three compartments of aspirin PK and *n* sets of two compartments describing COX-1 inactivation in MK units (cells born at a similar time *τ_i_*) and platelets generated from them. Further details can be found in our previous work [S1].

**Aspirin PK**

A linear three-compartment model is assumed to describe low dose aspirin PK. Model equations are:

| $\dot{A}_{S}\left( t \right)= {-\left( k_{1}+k_{d,S}+k_{he} \right)A_{S}\left( t \right)+k}_{2}A_{C}\left( t \right)+I(t)$ $A_{S}\left( 0 \right)=0$ | (S-1) |
| --- | --- |
| $\dot{A}_{B}\left( t \right)=-\left( k_{2}+k_{3}+k_{d,B} \right)A_{B}\left( t \right)+k_{1}A_{S}\left( t \right)+k_{4}A_{T}\left( t \right)$ $A_{B}\left( 0 \right)=0$ | (S-2) |
| $\dot{A}_{T}\left( t \right)=-\left( k_{4}+k_{d,T} \right)A_{T}\left( t \right)+k_{3}A_{B}\left( t \right)$ $A_{T}\left( 0 \right)=0$ | (S-3) |

where:

- *t* indicates time.
- *A_S_(t), A_B_(t), A_T_(t)* [ng] represent the time course of aspirin amounts in the portal circulation, systemic circulation and tissues, respectively. The dot indicates the time derivatives.
- *k_1_* and *k_2_* [min^-1^] represent the rate constants between the portal circulation and the systemic circulation; *k_3_* and *k_4_* [min^-1^] represent the rate constants between the systemic circulation and tissues. Numerical values for these parameters were derived from the physiology.
- *k_he_* [min^-1^] represents the aspirin hepatic extraction (HE) and is fixed equal to *k_1_*, to account for the ability of the liver to inactivate approximately 50% of absorbed aspirin**.**
- *k_d,T_, k_d,B_, k_d,S_* [min^-1^]account for the additional aspirin hydrolysis in blood and tissues, and are fixed as *k_d,T_ = k_d,B_ = k_d,S_* consistently with the whole body clearance of aspirin.

*I(t)* [ng/min] represents the rate of aspirin appearance in the systemic circulation. Since aspirin treatment is based on repeated daily dosing of enteric coated aspirin *I(t)* was modeled by properly combining the description of the aspirin appearance after a single dose, which is 4 hours delayed according to literature.

By normalizing aspirin amounts in the three compartments by their distribution volumes *V_S_, V_B_, V_T_* [ml], aspirin concentrations [*A_S_(t)*], [*A_B_(t)*], [*A_T_(t)*] [ng/ml] in, respectively, the portal circulation, systemic circulation and tissues are derived.

**COX-1 dynamics in a single MK unit**

During the maturation phase, lasting *T_1_*, COX-1 dynamics in a single MK unit of cells born at time *τ_i_* is described as the balance between input (i.e., *de novo* synthesis) and output (i.e., degradation and acetylation) fluxes, while during the platelet generation an additional output flux describes COX-1 transfer to platelets, lasting *T_2_*. Dynamic events such as COX-1 synthesis, degradation, and transfer to platelets depend upon the lag between time *t* and time *τ_i_*, so that model equations are:

|  | $p\left( t-\tau_{i} \right)-k_{d,x}{\left( t-\tau_{i} \right) x}_{MK}\left( t \right)-\varphi\left( {[x}_{MK}(t)],{[A}_{T}(t)] \right)$ | maturation:  ${t\in[\tau}_{i}, \tau_{i}+T_{1}]$ |  |
| --- | --- | --- | --- |
| $\dot{x}_{MK}\left( t \right)=$ |  |  | (S-4) |
|  | $p-k_{d,x}x_{MK}\left( t \right)-k_{5}\left( t-\tau_{i} \right)x_{MK}\left( t \right)-\varphi\left( {[x}_{MK}(t)],{[A}_{T}(t)] \right)$ | platelet generation:  $t\in[\tau_{i}+T_{1},\tau_{i}+T_{1}+T_{2}]$ |  |

where:

- *x_MK_(t)* [ng] and [*x_MK_(t)*]= *x_MK_(t)*/ *v_MK_(t)* [ng/ml] are the time course of COX-1 amount and concentration, respectively, inside a generic MK unit originated at time *τ_i_.* The volume *v_MK_(t)* was modeled using proper functions, to account for the exponential growth during maturation and the exponential decay during the platelet generation.
- *p(t-τ_i_)* [ng/min] and *k_d,x_(t-τ_i_)* [min^-1^] represent COX-1 input function from expression/translation and degradation rate constant, respectively. They were phenomenologically modeled in order to reproduce a fast COX-1 expression at the beginning of the megakaryocyte life, followed by a decline to a constant level in the remaining life of the megakaryocyte.
- $\varphi\left( {[x}_{MK}(t)],{[A}_{T}(t)] \right)=\frac{\lambda\left[ {[A}_{T}(t)]{\cdot[x}_{MK}(t)] \right]^{n}}{{(k_{m})}^{n}+\left[ {[A}_{T}(t)]{\cdot[x}_{MK}(t)] \right]^{n}}$ [ng/min] is COX-1 acetylation by aspirin, taking place in the MK unit, modeled as a sigmoidal function of the product of COX-1 and aspirin concentrations at time *t*, in keeping with a threshold-saturation mechanism proposed in literature.
- $k_{5}(t-\tau_{i})$ is the rate constant at which COX-1 leaves the MK unit compartment and is transferred to platelets during the final maturation stage, expressed in terms of the reduction in MK unit volume *v_MK_*.

**COX-1 dynamics inside the platelets formed from a single MK unit**

COX-1 dynamics in the platelets formed from a single MK unit result from the balance between the input flux from platelet generation, lasting *T_2_* and present only in the early phase, and two output fluxes the former due to acetylation, lasting *T_3a_*, and the latter to peripheral platelet destruction lasting *T_3b_*, so that model equations are:

| $\dot{x}_{P}\left( t \right)=$ | $k_{5}\left( t-\tau_{i} \right) x_{Mk}\left( t \right)-\varphi\left( [x_{p}\left( t \right)]{,0.75[A}_{B}\left( t \right)]+0.25{[A}_{S}(t)] \right)$ | platelet generation:  $t\in[\tau_{i}+T_{1},\tau_{i}{+T_{1}+T}_{2}]$ |  |
| --- | --- | --- | --- |
|  |  |  |  |
|  | $-\varphi\left( [x_{p}\left( t \right)]{,0.75[A}_{B}\left( t \right)]+0.25{[A}_{S}(t)] \right)$ | platelet activity:  $t\in[\tau_{i}+T_{1}+T_{2},$  $\tau_{i}+T_{1}+T_{2}+T_{3a}]$ | (S-5) |
|  |  |  |  |
|  | $-\varphi\left( [x_{p}\left( t \right)]{,0.75[A}_{B}\left( t \right)]+0.25{[A}_{S}(t)] \right)-k_{6}(t-\tau_{i}) x_{P}\left( t \right)$ | platelet death:  $t\in[\tau_{i}+T_{1}+T_{2}+T_{3a},$  $\tau_{i}+T_{1}+T_{2}+T_{3a}+T_{3b}]$ |  |

where:

- *x_p_(t)* [ng] and [*x_p_(t)*] = *x_p_(t)/ v_p_(t)*[ng/ml] are the time course of COX-1 amount and concentration in platelets derived from a single MK unit. The volume *v_p_(t),* increases during MK proliferation, parallel to the decrease of MK volume, remains constant during platelet life and then decreases to zero during their death phase.
- *φ*([*x_p_(t)*]*,0.75*[*A_B_(t)*] *+ 0.25*[*A_S_(t)*]) [ng/min] is the sigmoidal COX-1 acetylation function inside platelets modulated by a weighted sum of aspirin patterns in the systemic and portal blood, assuming a 75/25 ratio of systemic/portal blood volume.
- $k_{6}\left( t-\tau_{i} \right)$ [min^-1^] is the rate constant of COX-1 disappearance, due to their peripheral destruction.

**COX-1 dynamics at the whole-body level**

The time course of whole-body COX-1 is obtained by summing up the contributions of all platelets in the systemic circulation, originating from MK units that were born at a time *τ_i_* ranging from t-T_1_-T_3_ and t-T_1_ where T_1_ and T_3_ are the MK and platelet lifetime, respectively. This variable, denoted by X(t), can be expressed as:

| $X\left( t \right)=\sum_{\tau_{i}\in[t-T_{1}-T_{3}, t-T_{1}]} x_{p}(t-\tau_{i})$ | (S-6) |
| --- | --- |

where x_p_ represents the amount of COX-1 inside the platelets derived from the MKs born at a time *τ_i_*.

**Parameter values**

As detailed in ref S1, few key parameters were tuned in order to fit the experimental data, while other parameters were inferred from literature, e.g. PK parameters were derived by considering nominal values of aspirin clearance, blood flows and volumes. Table 1 shows the values of the most relevant model parameters defined in Fig 1, while we refer to the Supplementary Table 1 of our previous work^S1^ for the remaining ones.

**Table S1. Numerical values of investigated major parameters**

Parameters of COX-1 dynamics inside MKs and platelets are intended per MK unit and platelet derived from it.

| **Parameters** | **Value** | **Unit of measure** |
| --- | --- | --- |
| *k_1_* | 0.943 | *min^-1^* |
| *k_2_* | 0.314 | *min^-1^* |
| *k_3_* | 1.019 | *min^-1^* |
| *k_4_* | 0.398 | *min^-1^* |
| *k_he_* | 0.943 | *min^-1^* |
| *k_d,S_=k_d,B_=k_d,T_* | 0.015 | *min^-1^* |
| *k_m_* | 17.78 | *ng^2^/ml^2^* |
| *λ* | 2.4 e^-3^ (control); 14.4 (ET) | *ng/min* |
| *T_1_* | 5 (healthy); 1.5 (ET) | *days* |
| *p* | 7.5 e^-4^ | *ng/min* |
| *k_d,x_* | 5 e^-4^ | *min^-1^* |
| *N* | 1 (control), 6 (ET) | [-] |
| *T_3a_* | 7 (healthy); 6 (ET1 and ET2) | *days* |

**Datasets**

We report the original dataset (Table S1 and Table S2) used to tune our original model [S1] and the dataset shown in Fig. 8 (main manuscript) about a single severely obese patient (Table S3) [S2]. The serum TxB_2_ data reported Tables S1, S2, S3 are reported as percentage values, normalized by the serum TxB_2_ baseline.

**Table S1. Experimental data of serum TxB_2_ for a population of healthy subjects under 100 mg aspirin dosage for a week of treatment.**

| **Healthy subjects** | | | | | |
| --- | --- | --- | --- | --- | --- |
| **days** | 20.5 | 21 | 22 | 23 | 27 |
| **TxB2 %**  **(mean ± SD)** | 0.77±0.67 | 1.06±0.75 | 3.5±3.04 | 12.33±7.22 | 81.96±21.87 |

**Table S2. Experimental data of serum TxB_2_ suppression and recovery for two subjects under essential thrombocythemia (ET) treated with 50 and 100 mg of aspirin the first and second week (ET1) and with 50, 100 an d150 mg aspirin during the first, second and third week (ET2).**

|  | **ET1** | | | | | | | | |
| --- | --- | --- | --- | --- | --- | --- | --- | --- | --- |
| **days** | 2 | 4 | 6 | 13 | 14.5 | 16 | 18 | 20 | / |
| **TxB2 %** | 11.76 | 4.12 | 2.65 | 0.98 | 20 | 69.12 | 117.65 | 78.43 | / |
|  | **ET2** | | | | | | | | |
| **days** | 2 | 4 | 6 | 13 | 20 | 21.5 | 23 | 25 | 27 |
| **TxB2 %** | 38.46 | 41.03 | 17.95 | 11.54 | 3.42 | 28.21 | 89.74 | 115.38 | 143.59 |

**Table S2. Experimental data of serum TxB_2_ related to one severely obese patient.**

| **Severe obese subject** | | | |
| --- | --- | --- | --- |
| **hours** | **4** | **24** | **48** |
| **TxB2 %** | 13.3 | 7.0 | 15.56 |

**Reference**

**S**1. Giaretta A, Rocca B, Di Camillo B, Toffolo GM, Patrono C. In Silico Modeling of the Antiplatelet Pharmacodynamics of Low-dose Aspirin in Health and Disease. *Clin. Pharmacol. Ther.* 2017;102(5):823-831.

**S2.**  Petrucci G, Zaccardi F, Giaretta A, Cavalca V, Capristo E, Cardillo C, et al. Obesity is

associated with impaired responsiveness to once ‐ daily low ‐ dose aspirin and *in vivo*

platelet activation. J Thromb Haemost. 2019 Jun;17(6) 885–95.
